# Disordered hypothalamus-pituitary-ovary axis in heterotopic extraovarian sex cord-stromal proliferation: a case report of fallopian tube serous adenofibroma

**DOI:** 10.1186/s12905-023-02407-y

**Published:** 2023-05-09

**Authors:** Isabel Hsu, Li-Hsuan Lee, Leonard Hsu, Shee-Uan Chen, Chao-Chin Hsu

**Affiliations:** 1 Department of Obstetrics and Gynecology, National Taiwan University Hospital, Taipei, Taiwan; 2 Nepean Hospital, Kingswood, NSW Australia; 3 Douglass Hanly Moir Pathology, Sydney, NSW Australia; 4 Department of Obstetrics and Gynecology, National Cheng Kung University Hospital, Tainan, Taiwan; 5 Department of Obstetrics and Gynecology, Taipei Medical University Hospital, Taipei, Taiwan

**Keywords:** Fallopian tube serous adenofibroma, Extraovarian sex cord–stromal proliferation, Gonadotropin-resistant ovary syndrome, anti-Müllerian hormone, Follicle stimulating hormone

## Abstract

**Background:**

Fallopian tube serous adenofibromas are uncommon tumors of the female genital tract, only dozens of cases have ever been reported. Earlier study indicated that they might be derived from embryonic remnants of the Müllerian duct. Clinical presentation of these tumors is usually asymptomatic. Small cysts of 0.5–3 cm in diameter are mostly incidentally found at the fimbriae end, with coarse papillary excrescences lined by epithelial cells and connective tissue stroma without nuclear pleomorphism or mitosis.

**Case presentation:**

A 23-year-old woman with normal secondary sexual characters and 46, XX karyotype, presented to the gynecology clinic complaining of irregular menstrual cycles. Laboratory studies reported unique discrepancy of hormone levels; anti-Müllerian hormone (AMH): 6.05 ng/mL (The normal range of AMH is 1.70–5.63 ng/mL in women aged under 35 years old), follicle stimulating hormone (FSH): 31.9 mIU/mL (reference range: 3.85–8.78, follicular phase; 4.54–22.51, ovulatory phase; 1.79–5.12, luteal phase; 16.74-113.59, menopause), and luteinizing hormone (LH): 52.0 mIU/mL (reference range: 2.12–10.89, follicular phase; 19.18-103.03, ovulatory phase; 1.20-12.86, luteal phase; 10.87–58.64, menopause), mimicking gonadotropin-resistant ovary syndrome. The ultrasound reported a right adnexal cyst of 10.4 × 7.87 × 6.7 cm. Laparoscopic evaluation was performed; pathology revealed serous adenofibroma of the fallopian tube with ovarian stroma contents. Heterotopic extraovarian sex cord-stromal proliferations was most probable. The patient’s hormone levels returned to the reproductive status two weeks after surgery; FSH: 7.9 mIU/mL, LH: 3.59 mIU/mL,and AMH: 4.32 ng/mL. The patient’s menstrual cycles have resumed to normal for over two years after removal of the fallopian tube cyst.

**Conclusions:**

This case of fallopian tube serous adenofibromas presented a discrepancy of serum AMH and FSH mimicking gonadotropin-resistant ovary syndrome. The clinical picture derived from heterotopic extraovarian sex cord-stromal proliferation indicated a disordered hypothalamus-pituitary-ovary axis.

## Background

Fallopian tube tumors are relatively uncommon tumors of the female genital tract, among which serous adenofibromas are benign and very unusual [1–3]. Only dozens of cases have been reported since 1909 [4, 5]. Fallopian tube serous adenofibromas are mostly discovered incidentally during workup or surgery performed for other health issues [4, 5]. A review of literature of earlier cases with clinicopathological presentation of this tumor has recently been summarized [5, 6]. Clinical presentation of these tumors is usually asymptomatic, though a few cases present with symptoms of abdominal pain, palpable mass, vaginal bleeding and urinary or bowel symptoms [5, 6]. Small cysts of 0.5–3 cm in diameter are mostly found at the fimbriae end, with pathology presentation of coarse papillary excrescences lined by epithelial cells and connective tissue stroma without nuclear pleomorphism or mitosis [5, 6]. An earlier study indicated that they might be derived from embryonic remnants of the Müllerian duct [7]. Recent studies suggested that fallopian tube serous adenofibromas are heterotopic sex cord-stromal proliferations found in fimbriae, which arise from heterotopic ovarian tissue exposed during ovulation [8, 9].

Normal ovulatory cycle requires tightly integrated interactions between the hypothalamus, pituitary, and ovary. An elevation of follicle stimulating hormone (FSH) in women indicates diminished ovarian function, as there is an absence of negative feedback on the hypothalamus-pituitary from the very low secretion of ovarian estradiol, leading to persistent secretion of FSH from pituitary gland [10]. The clinical picture of gonadotropin-resistant ovary syndrome (ROS) is characterized by hypergonadotropic amenorrhea, normal secondary sexual characteristics, normal 46, XX karyotype, and age-appropriate anti-Müllerian hormone (AMH) values [11, 12]. ROS patients have morphologically normal ovaries with ordinary ovarian primordial follicles, which however are resistant to exogenous gonadotropin stimulation [13, 14]. Gonadotropin-resistant ovary syndrome is occasionally associated with autoimmune disease [15, 16], or antibodies against FSH receptor or mutations in FSH receptor gene [17–19].

We report the case of a woman of reproductive age with tubal serous adenofibroma who presented with irregular menstrual cycles and discrepant AMH and FSH levels that resembled characteristics of ROS. The patient resumed normal menstrual cycle and her serum hormone levels returned to reproductive status after removal of the fallopian tube cyst.

## Case presentation

**Fig. 1:**
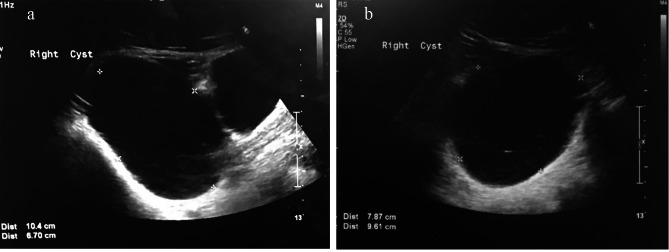
**(A)(B)** Abdominal sonographic scanning with size measurement of the mass

A 23-year-old woman, G0, reports irregular menstrual cycles for 6 months. Her body mass index (BMI) was 22.5 Kg/M^2^ and she presented with appropriate sex characteristics; Tanner stages 5 in both pubic hair and breast development. The patient‘smenarche occurred at the age of 12 years old and had relatively regular menstrual cycle intervals of 28–30 days and duration of 3–5 days. No heavy menstrual blood flow or blood clots were noted. No dysmenorrhea or symptoms of endometriosis were noted either. The acute onset of irregularity in the patient’s menstrual cycle occurred six months prior her first gynecologic visit. Her menstrual period was initially delayed for two weeks, then the entire menstrual cycle interval prolonged to two months. The patient then visited a gynecology clinic for further examination. No signs of androgen and/orcortisol excess, thyroid abnormalities, and galactorrhea, all of which might have resulted in the irregularity of menstrual periods, were noted. She denied of previous sexual exposure. The initial hormone profile blood test taken showed elevated FSH and luteinizing hormone (LH) of 31.1 mIU/mL and 52.0 mIU/mL (Table 1). An ultrasound scan was suggested, but the patient hesitated due to personal reasons. Blood test three months later showed FSH: 31.9 mIU/mL, LH: 17.22 mIU/mL, AMH: 6.05 ng/mL, and both estradiol and progesterone in ovulatory phase (Table 1), other hormone tests as listed in Table 1 were normal. The serum β-hCG level was not tested due to absence of sexual debut. Also, no intrauterine gestational sac or other symptoms and signs of ectopic pregnancy were noted. Tumor marker was within normal range; CA125: 9.51 U/ml. Karyotype analysis indicated normal 46, XX. No signs of polycystic ovarian syndrome (PCOS) such as hirsutism, acne, buffalo hump and obesity were noted. Ultrasound examination showed uterine size of 6.10 × 4.18 × 3.19 cm with endometrium thickness of 12 mm. Left ovary measured 2.81 × 1.81 cm. No multiple, small antral follicles distributed peripherally or throughout the dense stroma characteristics of PCOS was noted on bilateral ovaries. A 10.4 × 7.87 × 6.7 cm simple cyst at the right adnexal area was noted (Fig. 1). The cyst is anechoic and unilocular, with clear content and no internal blood flow. No septums,solid components, calcifications, papillary formations, and ascites were found under sonographic examination. A right ovarian cyst was highly suspected.

**Table 1:** Serum hormone profiles before and after surgical removal of fallopian serous adenofibroma

Date	^a^	^b^	^c^	^d^
AMH (ng/mL)		6.05	4.32	4.53
FSH (mIU/mL)	31.1	31.9	7.9	17.22
LH (mIU/mL)	52.0	17.22	3.59	7.55
E2 (pg/mL)	133.0	38.2	73.1	26.12
P4 (ng/mL)	0.69	0.38	3.43	3.43
Total T (ng/mL)		0.19	0.18	0.18
Free T (pg/mL)		1.93	1.85	1.85
DHEA-S (µg/dL)		55.2	53.5	61.0
Inhibin B (pg/mL)		74.7	91.7	83.9
Inhibin A (pg/mL)		31.6	29.2	49.2
PRL (ng/mL)	18.85			
CA-125 (U/mL)		9.51		10.90

^a^: 117 days before surgery; ^b^: 13 days before surgery and day 3 of the menstrual cycle; ^c^: 13 days after surgery and day 15 of the menstrual cycle; ^d^: 10 months after surgery and day 17 of the menstrual cycle

Abbreviations and reference range:

AMH (anti-Müllerian hormone): 1.70–5.63 ng/mL in women aged under 35 years old;

FSH (follicle stimulating hormone IU/mL): 3.85–8.78, follicular phase; 4.54–22.51, ovulatory phase; 1.79–5.12, luteal phase; 16.74-113.59, menopause;

LH (luteinizing hormone IU/mL): 2.12–10.89, follicular phase; 19.18-103.03, ovulatory phase; 1.20-12.86, luteal phase; 10.87–58.64, menopause;

E2 (estradiol pg/mL): 27–122, follicular phase; 95–433, ovulatory phase; 49–291, luteal phase;

P4 (progesterone ng/mL): 0.31–1.52, follicular phase; 5.16–18.56, luteal phase;

Total T (testosterone): 0.1–0.75 ng/mL in women aged 21–73 years old;

Free T (testosterone): 1.73–15.9 pg/mL in women aged 20–46 years old;

DHEA-S(dehydroepiandrosterone sulfate µg/dL): 20.1-414.2 in women aged 20–29 years old; 47.8–336 in women aged 30–39 years old

Inhibin A (pg/mL): 1.8–90.3, follicular phase; 16.9–91.8, ovulatory phase; 2.7–97.5, luteal phase;

PRL (prolactin ng/mL): 1.9–25

**Fig. 2:**
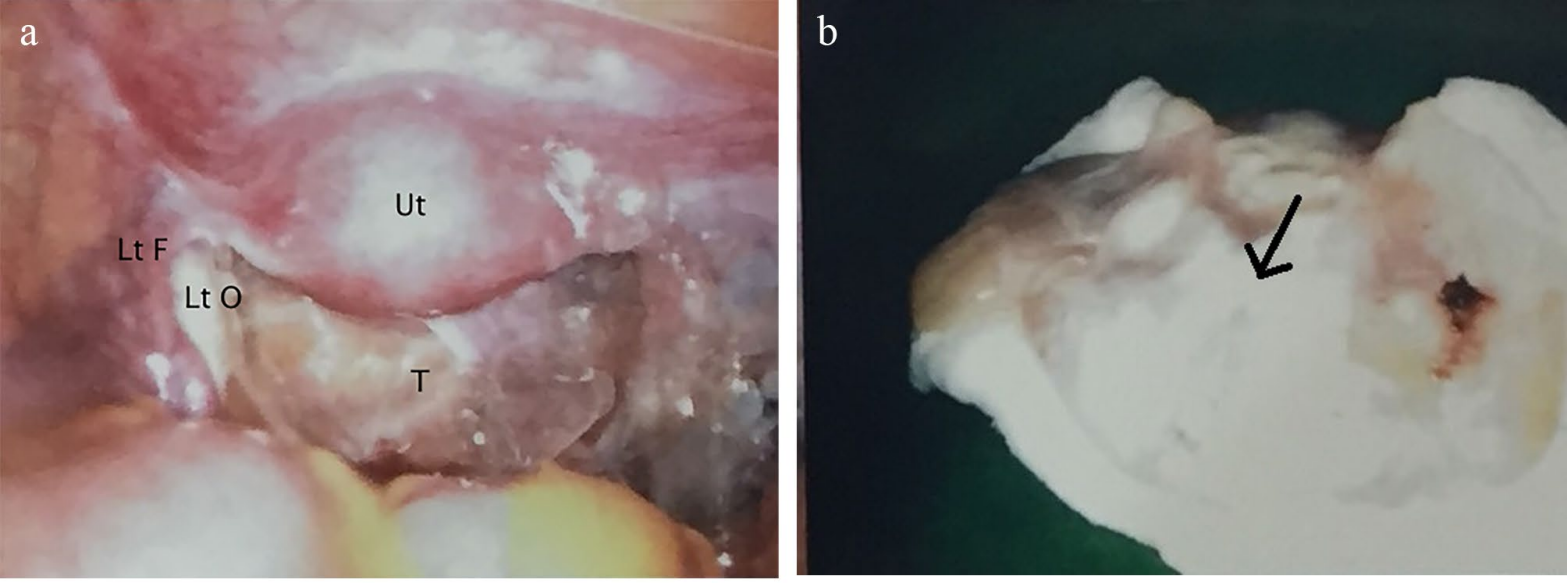
**(A)** The picture of pelvic cavity taken during the operation. Ut: uterus; T: an amorphous mass extended from right adnexa and occupied the entire cul-de-sac; Lt O: left ovary; Lt F: left fallopian tube. **(B)** gross picture of the right adnexal mass fixed in formalin. Arrow: polyps in inner wall of the mass

**Fig. 3:**
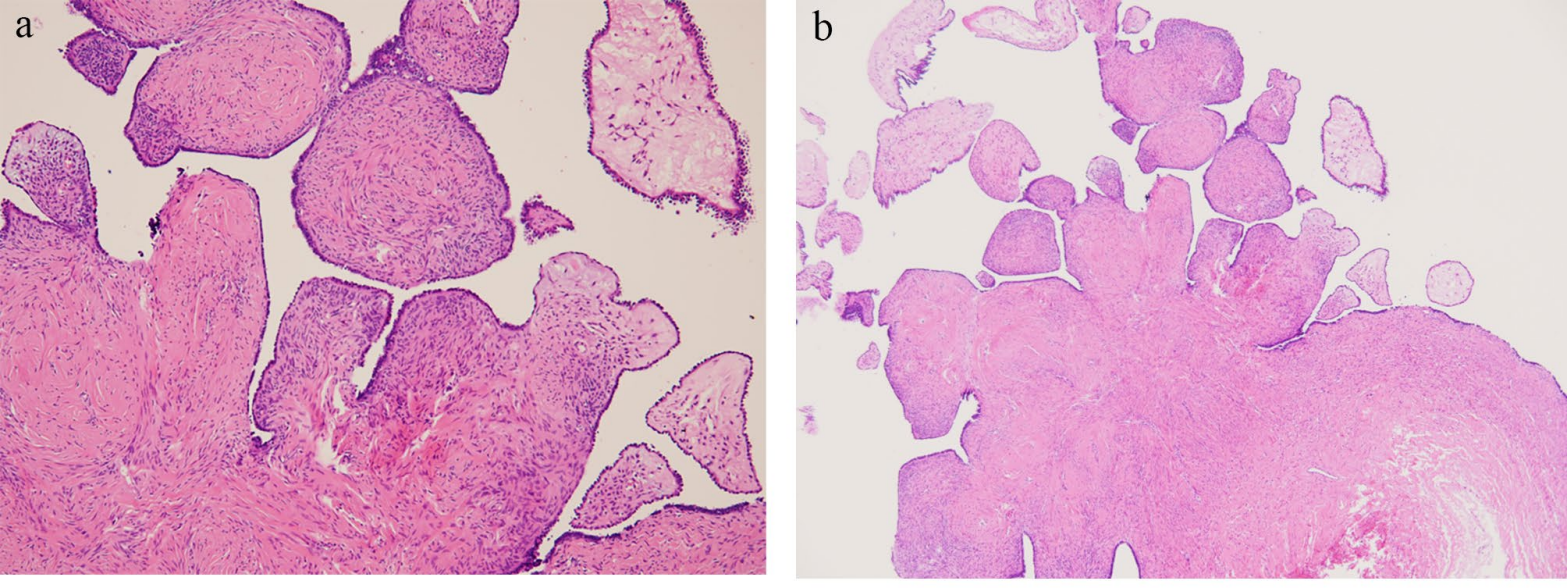
**(A)** 100X and **(B)** 200X. Hematoxylin and Eosin stain of the cyst. Serous adenofibroma of tubal cyst lined by low cuboidal to ciliated columnar cells was identified, beneath which spindle cells were present. Multiple rough intracystic papillary projections supported by fibrous tissue were observed

Laparoscopic evaluation was performed two weeks later. A right fallopian tube cyst was completely excised (Fig. 2A). During operation, the cyst occupied most of the right pelvic cavity. Though adjacent to the right ovary, there was no adhesion between the pelvic cyst and right ovary. Therefore, the removal of the pelvic cyst was performed smoothly and no ovarian tissue was resected during the operation. Bilateral ovaries were of normal size and were grossly normal under laparoscopic inspection. No characteristics of PCOS were noted, including thickened white capsule of the ovary. No other abnormal findings were noted over the pelvic region, including the uterus, left adnexa, cul-de-sac, and pelvic wall. The specimen removed consisted of one tissue fragment measuring 8.0 × 6.2 × 0.3 cm. Pathologic examination showed a cyst with smooth internal and external surface and eight polyps measuring up to 0.8 cm in maximal diameter connected to the inner wall (Fig. 2B). Microscopically, serous adenofibroma lined by simple flattened cuboidal epithelial cells with occasionally discernible apical cilia resting on fibrous wall was noted. Some areas showed broad and blunt papilla formation with attenuated epithelium and dense hypocellular fibrotic stroma (Fig. 3). No atypia or invasion was observed. Based on the histopathological findings, a pathologic diagnosis of serous adenofibroma of the fallopian tube was given. Focal area of ovarian stroma with corpora lutea was also noted.

The patient’s hormone levels returned to normal reproductive ranges two weeks after removal of the fallopian tube cyst, except for a slightly elevated FSH of 17.22 mIU/mL 10 months after the operation (Table 1). She has resumed normal menstrual cycles for over two years. Ultrasound scans were performed 10 and 18 months after the removal of the fallopian tube cyst: no ovarian or adnexal lesion was noted. The serum androgen levels were analyzed before and after the operation and no hyperandrogenism was noted (Table 1). The serum inhibin A and B concentrations were also in the range of reproductive status.

## Discussion and conclusions

Tubal adenofibromatous lesions have recently been regarded as extraovarian sex cord-stromal proliferations [9]. In the present case, focal area of ovarian stroma with corpora lutea was noted in tubal adenofibroma lesion. Thus, heterotopic extraovarian sex cord-stromal proliferation was likely. Heterotopic extraovarian sex cord-stromal proliferations have mostly been incidentally detected and most of which were measured 1–7 mm in size and located at the fimbrial end of the fallopian tube [8, 9, 20, 21]. The present case was unusual for its large size and only another three case presentation reported tubal adenofibroma with the size up to 8 cm [5, 6, 22]. The application of immunohistochemical markers and molecular analysis on extensive sampling of the specimen is mandatory to provide an accurate diagnosis of sex cord-stromal tumors [23–25]. The incipient adenofibromas (< 3 mm in size) were noted in 23% of high-risk patients undergoing risk-reducing surgery [26]. Fimbrial adenofibromas were found in 9.9% of cases and were more common in women at high risk of extrauterine high-grade serous carcinoma [20]. Moreover, sex cord elements proliferation of ovarian serous cystadenofibromas have been shown to attain estrogen-secreting activities in cases associated with uterine abnormalities [27, 28]. Though fallopian tube adenofibromas are basically non-functional, recent report on cases of fallopian tube adenofibromas showed sex cord proliferation resembling microscopic adult granulosa cell tumors [8]. An adult granulosa cell tumor characterized by abnormally elevated (8–10 times normal range) AMH levels, and hyperandrogenism has been noted [29]. It is likely that microscopic sex cord or granulosa cell components, both undetected in our initial examination, did exist and alter the patient’s hypothalamus-pituitary-ovarian axis, resulting in characteristics that mimicked ROS.

The menopausal level FSH, age-appropriate AMH, normal secondary sexual characters, and normal karyotype of this case are characteristics that mimic ROS, except for irregular menstrual cycles other than amenorrhea [14, 30]. Similar to the successfully resumed ovarian function in ROS patients who received ovarian biopsy [31, 32], our patient resumed normal menstrual cycles after removal of the fallopian tube cyst. One diagnostic criterion of ROS is the presence of primordial follicles in ovarian biopsy [13, 14]. However, the presence of ovarian follicles in our patient cannot be determined because we did not perform biopsy of the ovaries during laparoscopic evaluation [33]. Though histologic proof could not be made, the ovulatory level of serum estradiol detected and the resumption of normal menstrual cycle after removal of the adnexal lesion suggested ovaries of reproductive state in our patient. Premature ovarian insufficiency and polycystic ovarian syndrome was possible but not likely. In our patient, resumed regular menstrual cycles and age appropriate AMH can be used to exclude the possibility of premature ovarian insufficiency, in whom very low to undetectable AMH presented [34]. The polycystic ovarian syndrome was also not possible by the gross picture of bilateral ovaries during operation and the plasma levels of androgens and inhibin detected.

During normal menstrual cycle, FSH concentrations rarely exceed 10 IU/L in the follicular phase, and do not exceed 20 IU/L during the mid-cycle peaks of FSH [35–37]. A recent study showed higher FSH levels of 22.44, 17.22, 15.9, 15.64 IU/L were detected at LH surge of the menstrual cycles of same individuals [38]. Thus, FSH level of 17.22 IU/L observed at 10 months after the surgery in our patient could be due to the sample taken during preovulatory surge. However, the repeated detection of FSH levels over 30 IU/L before surgery was still higher than most physiological investigations. Four months prior to surgery, the patient’s LH and estradiol levels were 52.0 mIU/mL and 133 pg/mL, respectively, implying the start of LH surge in the preovulatory phase. The ovulatory level of serum estradiol was presumed to down-regulate FSH secretion from the pituitary gland [10], but our patient’s serum FSH level was as high as 31.1 mIU/mL (Table 1). One week before surgery, the patient’s hormone profile was still atypical for her age with serum FSH and LH levels of 31.9 mIU/mL and 17.22 mIU/mL, respectively. Nevertheless, the hormone levels returned to normal reproductive status two weeks after surgery (Table 1), suggesting that the hypothalamus-pituitary-ovary axis resumed normal function.

The fallopian tube has been receiving increased attention in gynecological oncology since considerable evidence suggests that it represents the site-of-origin of many pelvic serous carcinomas [39–45]. In a recent study on ovarian tumors, 52.2% of the fallopian tubes were normal and 39.2% were affected by the tumors. Amongst grossly normal fallopian tubes, almost 70% were histologically normal, while transitional metaplasia was present in 17.4%, endometriosis in 8.1%, and adenofibroma in 2.2% [46]. Differential diagnosis of tumors of tubal origin includes tubal carcinoma, serous papillary tumor of low malignant potential (STLMP), and serous borderline tumor. STLMP and serous borderline tumors are characterized by cellular pleomorphism and nuclear atypia [47, 48], which were not observed in our case. Recent studies have demonstrated that most so-called ovarian high-grade serous carcinomas are likely to arise from the epithelium of the distal fimbrial portion of the fallopian tube from a precursor lesion known as serous tubal intraepithelial carcinoma (STIC) [49]. Fallopian tube epithelium that implants on the ovary, is suggested to be the origin of low-grade and high-grade serous carcinoma [50]. Besides, fallopian tube adenofibromas are seen in 10% of women with *BRCA 1/2* mutations or a strong family history of breast/ovarian carcinomas, but in only 2.5% of non-high-risk women [51]. Thus, fallopian tube tumors, whether benign or malignant, deserve close attention. Regarding young women suffering from pelvic mass requiring resection of the ovaries and/or fallopian tubes due to either neoplasm or heterotopic pregnancy, counselling should foster access to fertility preservation procedures [52, 53]. Moreover, the management of pelvic cyst/neoplasm requires a holistic approach focused on reducing overall inflammation, increasing detoxification with the introduction of antioxidant vitamins, the influence of lifestyle including diets and various nutritional factors, and attenuating troublesome symptoms [54].

There are limitations in this case presentation which included [1] the resected specimen was not preserved for further immunohistochemistry or molecular analysis; [2] not enough serum was preserved for detailed analysis of hormone profiles and no duplication of analysis could be done; [3] the blood taken for the examination was not scheduled and the possible circadian variations and fluctuations of hormone concentrations throughout the menstrual cycle could not be properly adjusted [4] the hormone profiles were assayed on the day blood samples taken, thus analytical variability and confounding factors could not be waived as four different days of analysis. In conclusion, this tubal adenofibromatous lesion presented an unusual big size of heterotopic extraovarian sex cord-stromal proliferation and discrepant serum FSH and AMH levels mimicking ROS. Whether the tubal serous adenofibroma of the patient possesses gonadotropin- or estrogen-secreting properties such as sex cord components, that may have interrupted or modulated the hypothalamus-pituitary-ovary axis remains unknown.

## Data Availability

All data generated or analysed during this study are included in this published article.

## List of abbreviations

FSH: Follicle stimulating hormone
ROS: Gonadotropin-resistant ovary syndrome
AMH: Anti-Müllerian hormone
BMI: Body mass index
LH: Luteinizing hormone
PCOS: Polycystic ovarian syndrome
STLMP: Serous papillary tumor of low malignant potential
STIC: Serous tubal intraepithelial carcinoma
DHEA-S: Dehydroepiandrosterone sulfate
PRL: Prolactin
E2: Estradiol
P4: Progesterone
T: Testosterone
